# Development of a brief assessment and algorithm for ascertaining dementia in low-income and middle-income countries: the 10/66 short dementia diagnostic schedule

**DOI:** 10.1136/bmjopen-2015-010712

**Published:** 2016-05-25

**Authors:** Robert Stewart, Maëlenn Guerchet, Martin Prince

**Affiliations:** King's College London (Institute of Psychiatry, Psychology and Neuroscience), London, UK

**Keywords:** Low- and middle-income countries, Diagnostic interview

## Abstract

**Objectives:**

To develop and evaluate a short version of the 10/66 dementia diagnostic schedule for use in low-income and middle-income countries.

**Design:**

Split-half analysis for algorithm development and testing; cross-evaluation of short-schedule and standard-schedule algorithms in 12 community surveys.

**Settings:**

(1) The 10/66 pilot sample data set of people aged 60 years and over in 25 international centres each recruiting the following samples: (a) dementia; (b) depression, no dementia; (c) no dementia, high education and (d) no dementia, low education. (2) Cross-sectional surveys of people aged 65 years or more from 12 urban and rural sites in 8 countries (Cuba, Dominican Republic, Peru, Mexico, Venezuela, India, China and Puerto Rico).

**Participants:**

In the 10/66 pilot samples, the algorithm for the short schedule was developed in 1218 participants and tested in 1211 randomly selected participants; it was evaluated against the algorithm for the standard 10/66 schedule in 16 536 survey participants.

**Outcome measures:**

The short diagnostic schedule was derived from the Community Screening Instrument for Dementia, the CERAD 10-word list recall task and the Euro-D depression screen; it was evaluated against clinically assigned groups in the pilot data and against the standard schedule (using the Geriatric Mental State (GMS) rather than Euro-D) in the surveys.

**Results:**

In the pilot test sample, the short-schedule algorithm ascertained dementia with 94.2% sensitivity. Specificities were 80.2% in depression, 96.6% in the high-education group and 92.7% in the low-education group. In survey samples, it coincided with standard algorithm dementia classifications with over 95% accuracy in most sites. Estimated dementia prevalences in the survey samples were not consistently higher or lower using the short compared to standard schedule.

**Conclusions:**

For epidemiological studies of dementia in low-income and middle-income settings where the GMS interview (and/or interviewer training required) is not feasible, the short 10/66 schedule and algorithm provide an alternative with acceptable levels of performance.

**Strengths and limitations of this study**

- The short 10/66 diagnostic schedule and algorithm were developed using original 10/66 pilot data from 25 international centres, using separate development and test data sets, allowing validation against known-group status.
- The short schedule and algorithm were further validated against the output from the standard 10/66 algorithm in international survey data from over 16 000 participants.
- The short schedule and algorithm involved the use of a depression screening scale rather than a diagnostic instrument, and there was thus a loss of specificity for dementia ascertainment in people with depression.
- 10/66 pilot samples are limited by their small individual sizes and selected nature; 10/66 survey samples were drawn from geographic catchments, so national representativeness cannot be assumed.

## Introduction

The global challenge of rising dementia prevalence is well recognised, as is the fact that this will be most apparent in low-income and middle-income countries because of rapid population ageing.^1^ Epidemiological research to investigate dementia prevalence, aetiology and impact requires a robust means to ascertain the diagnosis in community surveys; however, most diagnostic schedules developed in high-income settings are strongly biased by educational attainment and do not have established cross-cultural validity. The 10/66 consortium was set up to redress the imbalance in dementia research between higher and lower income settings,^2^ and it began this process by assembling appropriate culture-fair instruments and developing a diagnostic algorithm to ascertain dementia status comparably across world regions.^3^ These initiatives in turn supported what has been the most extensive programme to date of cross-sectional and longitudinal research in community populations of older people including rural and urban catchment surveys in 12 countries, typically involving samples of 1000–2000 participants per site.^4^

The instruments contributing to the 10/66 diagnostic schedule and algorithm are the Community Screening Instrument for Dementia (CSI-D),^5^ ^6^ the CERAD 10-word list recall task^7^ and the Geriatric Mental State (GMS) interview,^8^ the last of which (through the AGECAT algorithm) primarily ascertains depression, among other mental disorders. The 10/66 algorithm, previously published, draws on the output of these instruments and applies a series of regression coefficients to assign a probabilistic diagnosis of dementia.^3^ However, although widely used in international research, the GMS interview generally takes 20–40 min to administer and requires a 2-day to 3-day dedicated interviewer training course. This potentially limits the application of the 10/66 schedule and algorithm in sites and situations where time is more limited for interviewer training and/or dementia assessment. With this in mind, we sought to develop and evaluate a shorter dementia assessment schedule using scores from the Euro-D scale in place of GMS–AGECAT output. The Euro-D is a 12-item depression screening scale, which was originally developed for international research and has been widely applied and evaluated.^9–12^ It is derived from individual GMS items—that is, can be extracted from studies that have used the GMS, and can be administered in its own right as a relatively brief (3–5 min) assessment. This paper reports our investigation of the derivation and validity of this ‘short’ 10/66 diagnostic schedule and algorithm.

## Methods

The questionnaires making up the 10/66 short diagnostic schedule (CSI-D cognitive assessment and informant interview, word list recall task and Euro-D) are displayed in online supplementary appendix 1 and are available from the 10/66 Dementia Research Group website (https://www.alz.co.uk/1066/resources.php) along with all data-processing algorithms.

10.1136/bmjopen-2015-010712.supp1 Supplementary appendix 1

Since the 10/66 short schedule is a subset of the standard schedule (substituting the Euro-D for the longer GMS but keeping all other components), the development of its diagnostic algorithm used existing data from studies in which the standard schedule had been administered. There were, in summary, two validation steps for the output of the short schedule and algorithm: first, against known-group status, using data from the 10/66 pilot studies; second, against output from the standard diagnostic schedule and algorithm, using data from a series of 10/66 cross-sectional surveys.

First, the short-schedule algorithm was developed and evaluated using data from the 10/66 pilot project. The pilot project has been described in detail previously,^3^ but in summary comprised the recruitment of 2885 participants aged 60 years and over from 25 centres in India, China and southeast Asia, Latin America and the Caribbean, and Africa. Each centre specifically recruited people with diagnosed dementia (n=729 across all sites) and three groups of people in whom dementia had been excluded: people with depression (n=702), with high education (n=694) and with low education (n=760). All pilot project participants and informants were interviewed blind to the allocation groups, and these interviews included the administration of the standard 10/66 schedule: CSI-D (the cognitive and informant interviews), GMS and 10-word list-learning task. For short-schedule algorithm development, Euro-D component items were extracted from the GMS data sets and scaled. A split-half technique was used for short-schedule algorithm development and initial validation, composed of the following steps, mirroring the original methodology used for developing the standard-schedule algorithm:^3^

1. A random 1413 participants were identified from the pilot study database for short-schedule algorithm development; of whom, 1218 had sufficient data for this.
2. A separate random sample of 1380 pilot study participants was identified for algorithm testing; of whom, 1211 had sufficient data.
3. Euro-D scores were generated from responses to the relevant individual GMS questions and were grouped by quartiles into four categories (0, 1–2, 3–5 and 6–12).
4. A calibration regression model was generated by entering this Euro-D categorical variable, together with standard categories from the CSI-D cognitive score, CSI-D informant score and 10-word list delayed recall. Regression coefficients were extracted and applied as multipliers for these score categories so that an overall individualised ‘algorithm score’ was generated for each participant in the development and test samples.
5. Receiver operating characteristics (ROC) curves were calculated for dementia as an outcome against these algorithm scores. An optimal cut-off on the final algorithm score was defined to categorise the presence or absence of dementia in the development sample.
6. Using the cut-off defined above, its distribution in the test sample (n=1211) was described and cross-tabulated against the four pilot study sampling groups (dementia; depression, no dementia; high education, no dementia and low education, no dementia). Its sensitivity was thus calculated as the proportion of people with dementia correctly classified, and its specificity was calculated for each of the three non-dementia groups.

The performance of the short-schedule algorithm was then further evaluated in the data set (release V.3_2) from the first wave of 10/66 surveys carried out at 12 urban and rural sites in eight countries (Cuba, Dominican Republic, Peru, Mexico, Venezuela, India, China and Puerto Rico). These have been described in detail previously,^4^ ^13–15^ but in summary comprised a combined sample of 16 536 participants aged 65 years and over recruited across these sites. The output for the short-schedule and standard-schedule algorithms was calculated for all participants, and dementia/non-dementia categories were cross-tabulated for the two algorithms at each site to estimate agreement. Finally, dementia prevalence estimates from both schedules were calculated and compared at each site in order to evaluate whether the short-schedule algorithm resulted consistently in overestimate or underestimate. SPSS software (version 22) was used for all analyses.

## Results

As described, the short-schedule algorithm was developed using data from a random 1413 participants in the 10/66 pilot samples; of whom, 1218 had sufficient source data for algorithm development. The calibration model derived from this data set is displayed in table 1. The area under the ROC curve (AUROC) statistic for the model as a predictor of dementia status was 0.972 (0.963 to 0.981) in the development data set, and a cut-point of 0.20+ on the predictor coefficient was identified as optimal. Applying this to the test set, the corresponding AUROC was 0.971 (0.961 to 0.981), and further data on the discriminability of the predictor coefficient are displayed in table 2. In summary, the short-schedule algorithm correctly classified 94% of dementia cases in the test data set (which contained 1211 participants with sufficient data for algorithm application), with ‘false-positive’ identification in 20% of cases with depression only, and in 3% and 7% of people without dementia in the high-education group and low-education group, respectively. The syntax used for short-schedule algorithm generation is displayed in online supplementary appendix 2.

10.1136/bmjopen-2015-010712.supp2 Supplementary appendix 2

**Table 1:** Calibration model (logistic regression) derived from the development database (n=1218)

	OR (95% CI)	β Coefficient
Euro-D		
0	1.0	0.0
1–2	1.8 (0.6 to 5.1)	0.576
3–5	0.7 (0.3 to 2.1)	−0.312
>5	0.3 (0.1 to 0.8)	−1.214
Word list recall		
7–10	1.0	0.0
5–6	4.5 (0.4 to 47.9)	1.500
4	5.6 (0.5 to 61.6)	1.721
1–3	11.6 (1.2 to 115)	2.454
0	25.5 (2.5 to 261)	3.241
CSI ‘D’ COGSCORE (cognitive assessment score)		
>31.84	1.0	0.0
30.67–31.83	1.0 (0.2 to 5.9)	−0.048
28.62–30.66	3.2 (0.7 to 15.9)	1.174
23.70–28.61	9.1 (2.0 to 42.3)	2.208
0–23.69	44.3 (8.9 to 222)	3.792
CSI ‘D’ RELSCORE (informant assessment score)		
0	1.0	0.0
0.5–1.5	4.5 (0.4 to 50.0)	1.497
2–5	9.5 (1.1 to 84.6)	2.251
5.5–12	76.9 (9.1 to 650)	4.343
>12	441 (50.2 to 3864)	6.088

**Table 2:** Performance of the 10/66 short-form dementia diagnosis algorithm in development and test data sets

	Sampling group			
	Dementia	Depression, no dementia	High education, no dementia	Low education, no dementia
Development data set				
N	302	285	313	318
Short-form case (%)	93.0	18.9	4.8	8.2
Test data set				
N	292	283	295	341
Short-form case (%)	94.2	19.8	3.4	7.3

The short-schedule algorithm was then applied in the 10/66 survey samples and compared with standard-schedule algorithm classifications at each site; the results of which are displayed in table 3. Estimated dementia prevalences at each site according to the two schedules and algorithms are displayed in table 4. In summary, disagreement levels between the algorithms were relatively low, and <5% for most sites. Where disagreements occurred, the short-form algorithm was more likely to identify a case in Latin American sites and less likely in the two Indian sites. However, estimated dementia prevalences were generally similar between the two algorithms with no consistent pattern of overestimation or underestimation by the short-schedule version.

**Table 3:** Comparison of the short-form and standard dementia diagnosis algorithms in 10/66 survey samples

	Number (%)					κ (SE)
	Total	Short-form and standard both negative	Short-form positive, standard negative	Short-form negative, standard positive	Short-form and standard both positive	
Cuba	2861	2539 (88.7)	54 (1.9)	21 (0.7)	247 (8.6)	0.85 (0.02)
Dominican Republic	1992	1700 (85.3)	76 (3.8)	33 (1.7)	183 (9.2)	0.74 (0.02)
Peru, urban	1332	1228 (92.2)	18 (1.4)	12 (0.9)	74 (5.6)	0.82 (0.03)
Peru, rural	546	500 (91.6)	12 (2.2)	10 (1.8)	24 (4.4)	0.66 (0.07)
Venezuela	1942	1783 (91.8)	35 (1.8)	14 (0.7)	110 (5.7)	0.80 (0.03)
Mexico, urban	996	881 (88.5)	34 (3.4)	14 (1.4)	67 (67.3)	0.71 (0.04)
Mexico, rural	987	880 (89.2)	30 (3.0)	11 (1.1)	66 (6.6)	0.74 (0.04)
Puerto Rico	1900	1735 (91.3)	25 (1.3)	10 (0.5)	130 (6.8)	0.87 (0.02)
China, urban	1126	1068 (94.8)	9 (0.8)	4 (0.4)	45 (4.0)	0.87 (0.04)
China, rural	974	934 (95.9)	2 (0.2)	7 (0.7)	31 (3.2)	0.87 (0.04)
India, urban	992	908 (91.5)	13 (1.3)	32 (3.2)	39 (3.9)	0.61 (0.05)
India, rural	888	850 (95.7)	20 (2.3)	38 (4.3)	43 (4.8)	0.57 (0.06)

**Table 4:** Dementia prevalence across 10/66 survey sites according to the short-form and standard diagnostic algorithms

	Number	% missing data	Dementia prevalence (%)	
			Short-form algorithm	Standard algorithm
Cuba	2944	2.8	10.5	10.8
Dominican Republic	2011	0.9	13.0	11.7
Peru, urban	1381	3.5	6.9	9.4
Peru, rural	552	1.1	6.6	6.5
Venezuela	1997	2.8	7.5	7.1
Mexico, urban	1003	0.7	10.1	8.6
Mexico, rural	1000	1.3	9.7	8.5
Puerto Rico	2009	5.4	8.2	11.7
China, urban	1160	2.9	4.8	7.0
China, rural	1002	2.8	3.4	5.6
India, urban	1005	1.3	5.2	7.5
India, rural	999	4.8	6.6	10.6

## Discussion

Using data from a large collection of pilot samples and a series of community surveys carried out in a range of low-income and middle-income countries from different world regions, we sought to develop a relatively brief dementia diagnostic assessment and algorithm for use in international epidemiological research. The objective was not to replace the standard 10/66 dementia diagnostic schedule and algorithm, which are already in wide international use, but to investigate the applicability of an alternative schedule for studies where GMS training for interviewers is not feasible, and/or where there is insufficient interview time for administering the GMS instrument itself. The short-form schedule itself would be expected to take 10–15 min with the participant and 5–10 min with an informant, and training sessions on these instruments can be comfortably accommodated within 1 day, although as with all epidemiological assessments, interviewer supervision and data quality monitoring are of paramount importance to achieve consistency.

The sensitivity of the short-schedule algorithm against clinically diagnosed dementia in the pilot samples was 94%, and its specificity was 80% in depression, 97% in people with high education and 93% in people with low education. These compare with respective performances of 94%, 85%, 97% and 94% previously published for the standard 10/66 schedule and algorithm.^3^ The comparable sensitivity for dementia, and specificities in high-education group and low-education group, is likely to reflect the salience of the CSI-D and word list recall components contributing to both algorithms; specifically, the CSI-D informant interview is recognised to be particularly important for reducing educational bias.^3^ A loss of specificity in people with depression is understandable, given that a diagnostic assessment for mental disorder in the standard schedule (GMS) is replaced with a relatively brief screening instrument (Euro-D) in the short version. However, it is encouraging that this loss is relatively small (5%) and without compromised sensitivity.

Although derived from a range of sites and settings, the 10/66 pilot samples are limited by their small individual sizes and selected nature. However, it was felt to be most appropriate to use these data for the generation of the short-schedule algorithm, just as they were used to develop the standard algorithm. Development data were supplemented with a cross-evaluation of the two schedules and algorithms using data from a series of community surveys.^13^ In this large data set, the short-schedule algorithm performed well against the standard version, with accuracy levels of above 95% in most sites and no evidence of substantial or consistent overestimation or underestimation of dementia prevalence. Specifically, considering previously published data on depression prevalence for 10/66 surveys in Mexico, Peru and Venezuela,^12^ there was no relationship between high/low prevalence sites and overestimation or underestimation of dementia using the short-form algorithm. Considering limitations, it is important to bear in mind that survey samples were derived from geographic catchments and national representativeness cannot be assumed; there were also a limited number of languages represented and wider international generalisability cannot be assumed. Additionally, the objective for the standard and short schedules and algorithms is to provide a probabilistic estimation of dementia rather than to apply diagnostic criteria. No grading of dementia severity is generated from these schedules, although supplementary items in the 10/66 surveys have been used for this purpose.

Taken together, our findings suggest that the 10/66 short dementia diagnostic schedule and algorithm have potential utility for epidemiological research where the standard interview schedule is not feasible. Some incorrect classification of dementia in people with depressive disorders is possible and should be borne in mind when interpreting findings; however, this does not appear to have a substantial influence on observed dementia prevalence. The primary purpose of the 10/66 programme has been to generate epidemiological research evidence on dementia prevalence, incidence and impact in low-income and middle-income countries to correct the evidence gap. The 10/66 diagnostic schedules and algorithms, short or standard, are therefore primarily designed for use in cross-sectional or prospective epidemiological research. They ought to have comparable utility in clinical research in these settings, although, to the best of our knowledge, they have not been applied in this way. Their utility in research in institutional settings or in clinical practice has not yet been evaluated.
