# Supplementary material for: Development of a brief assessment and algorithm for ascertaining dementia in low-income and middle-income countries: the 10/66 short dementia diagnostic schedule
Source: BMJ Open. 2016 May 25;6(5):e010712. doi: 10.1136/bmjopen-2015-010712 (PMC4885443; doi:10.1136/bmjopen-2015-010712)
Supplement: Supplementary appendix 1 [file bmjopen-2015-010712supp1.pdf]

Appendix 1 - components of the 10/66 short dementia diagnostic schedule

See also https://www.alz.co.uk/1066/resources.php for further 10/66 measures and data-processing algorithms

10/66 Dementia Research Group
Population-based study
May 2003

COGNITIVE ASSESSMENTS

(Word list recall and CSI-D)

WORD LIST LEARNING

I am going to read out a list of words. Please listen carefully, as I will ask you to repeat them back to me when I have finished.

Read out the ten words, pausing for one second between each. Tick correct words on the grid below

	1ST	2ND	3RD	
BUTTER				BUTTER
ARM				ARM
LETTER				LETTER
QUEEN				QUEEN
TICKET				TICKET
GRASS				GRASS
CORNER				CORNER
STONE				STONE
BOOK				BOOK
STICK				STICK
**TOTAL SCORE**				

1st trial {LEARN1}

1. Now please tell me all the words you can remember

Interviewer - Score total number of words correctly recalled in the box above

2nd trial {LEARN2}

2. Thank you. Now I will read out the words to you one more time. Again, please listen carefully, as I will ask you to repeat the words when I have finished.

Interviewer - Read out the ten words, pausing for one second between each.

Now please tell me all the words you can remember

Interviewer – Score total number of words correctly recalled in the box above

3rd trial {LEARN3}

3. Thank you. Now I will read out the words to you one last time. Again, please listen carefully, as I will ask you to repeat the words when I have finished.

Interviewer - Read out the ten words, pausing for one second between each.

Now please tell me all the words you can remember

Interviewer - Score total number of words correctly recalled in the box on the previous page

The Community Screening Instrument for Dementia (CSI-D)

4. {NAME}

I'd like you to remember my name.
My last name is xxxxxxxxx . Can you repeat that please ?

Cannot repeat name	0
Successfully repeats name	1

WE WILL BEGIN WITH NAMING THINGS. I WILL POINT TO SOMETHING AND I WOULD LIKE YOU TO TELL ME THE NAME OF THE OBJECT. FOR EXAMPLE

5. {PENCIL}

(Interviewer shows a pencil)

What is this called?

Incorrect	0
Correct	1

6. {WATCH}

(Interviewer points to their watch)

What is this?

Incorrect	0
Correct	1

7. {CHAIR}

(Interviewer pats chair)

What about this?

Incorrect	0
Correct	1

8. {SHOES}
(Interviewer points to shoes [or socks or stockings if they have left shoes outside])
And these?

Incorrect	0
Correct	1

9. {KNUCKLE}
(Interviewer shows their knuckles)
What do we call these?

Incorrect	0
Correct	1

10. {ELBOW}
(Interviewer points to their elbow)
What do we call this?

Incorrect	0
Correct	1

11. {SHOULD}
(Interviewer points to their shoulder)
What do we call this?

Incorrect	0
Correct	1

I WAS JUST SHOWING YOU THINGS AND YOU TOLD ME WHAT WE CALL THEM. NOW I WILL TELL YOU THE NAME OF SOMETHING AND I WANT YOU TO DESCRIBE WHAT IT IS. FOR EXAMPLE

12. {BRIDGE}
What is a bridge?

Incorrect	0
Correct*	1

*Correct answers: to walk across water, to climb up etc.

13. {HAMMER}
What do you do with a hammer?

Incorrect	0
Correct*	1

*Correct answers: to drive a nail into something

14. {PRAY}
What do people do in a church/ temple/ mosque(as appropriate)

Incorrect	0
Correct*	1

*Correct answers: to pray, to wed

15. {CHEMIST}

<u>Where do we go to buy medicine</u>

	Incorrect	0
	Correct*	1

*Correct answers: chemist, pharmacy etc (accept locally appropriate answers)

16. {REPEAT}

<u>Now I would like you to repeat what I say</u>

(<u>Only one</u> presentation is allowed, so the interviewer must read the phrase clearly and slowly enunciating carefully)

<u>'No ifs, ands or buts'</u>

Incorrect	0
Correct	1
(exact phrase only)	

Notes for translators –
Ganguli's Hindi MMSE points out that the phrase in English consists entirely of 'functor' words, prepositions and conjunctions used as nouns. This is apparently a test of verbal fluency which patients with non-fluent aphasia find particularly difficult to repeat. Your challenge is then to find an equivalent. In the Hindi MMSE Ganguli says she settled for the Hindi for 'neither this nor that' but doesn't give the Hindi phrase in the paper

WORD LIST LEARNING - delayed recall

Do you remember that I read out to you a list of words? How many of those words do you remember now? Could you please tell me all the words you can remember.

Interviewer - Score correct words in the grid below

BUTTER	
ARM	
LETTER	
QUEEN	
TICKET	
GRASS	
CORNER	
STONE	
BOOK	
STICK	
**TOTAL SCORE**	

17. {RECALL} ___

Total number of words correctly recalled

18. {NRECALL}
Do you remember my name? What is it?

Incorrect	0
Correct	1
(allow minor errors)	

ANIMAL NAMING

Now we are going to do something a little different, I am going to give you a category, and I want you to name, as fast as you can, all of the things that belong in that category. For example, if I say 'articles of clothing' you could say shirt, tie or hat. Can you think of other articles of clothing.

Wait for the subject to give two words. If the subject succeeds, indicate that the responses were correct and proceed to the test itself. If the subject gives an inappropriate word or reply, correct the response and repeat the instructions. If it becomes clear that the subject still does not understand the instruction, terminate this task and explain why this is so. After you are satisfied that the subject understands the task, and has given two words naming articles of clothing, say:

That's fine. I want you to name things that belong to another category, 'animals'. I want you to think about all the many different kinds of animal you know. Think of any kinds of animal in the air, on land, in the water, in the forest, all the different animals. Now I would like for you to tell the names for as many different animals as you can. You will have a minute to do this. Are you ready? Let's begin.

Allow one minute precisely. If the subject stops before the end of the time, encourage them to try to find more words. If they are silent for 15 seconds repeat the basic instruction ('I want you to tell me all the animals you can think of'). No extension on the time limit is made in the event that the instruction has to be repeated.

(The score is the sum of acceptable animals. Any member of the animal kingdom, real or mythical is scored correct, except repetitions and proper nouns. Specifically, each of the following gets credit: a species name and any

accompanying breeds within the species; male, female and infant names within the species)

19. {ANIMALS} _________

<u>Now I am going to tell you three words and I would like you to repeat them after me</u>

<u>Boat</u>
<u>House</u>
<u>Fish</u>

Interviewer - Score one point for each correct word on first attempt

20.1 FIRST TRIAL SCORE {WORDIMM}

No words remembered	0
1 word remembered	1
2 words remembered	2
3 words remembered	3

Then go on to repeat the three words, up to a total of six times until the subject has remembered them all correctly

20.2 RECORD NUMBER OF TRIALS UNTIL REPEATED {TRIALNO} SUCCESSFULLY (i.e. '1' if repeated correctly first time) _______

<u>Very good, now try to remember these words because I will be asking you later</u>

21. {TOWN}
<u>What is the name of this city/town/village?</u>

Incorrect	0
Correct	1

22. {CHIEF}
<u>What is the name of the mayor / village head?</u>

Incorrect	0
Correct	1

23. {STREET}
<u>What are the names of two main streets near here?</u>
Or (if inappropriate) What is the name of a river near here?

Incorrect	0
Correct	1

24. {STORE}

<u>Where is the local market / local store?</u>

Incorrect	0
Correct	1

25. {ADDRESS}

<u>What is your address?</u>

Or (if inappropriate) Who lives next door?

Incorrect	0
Correct	1

26. {WORDDEL}

<u>Do you remember the three words I told you a few minutes ago?</u>

No words remembered	0
1 word remembered	1
2 words remembered	2
3 words remembered	3

27. {LONGMEM}

Construct a locally appropriate equivalent of:
USA: What is the name of the civil rights leader who was assassinated in Memphis in 1968?
Nigeria: Who was the military leader of the Ibos during the Nigerian Civil war fought between 1967-1970?

Incorrect	0
Correct	1
Doesn't know	9

The key to this is to give the participant the date and the event and ask them for the identity of the famous person who was involved. The event should be so well known that practically no non-demented person should get it wrong!

<u>Now I would like to ask some questions about time</u>

28. {MONTH}

<u>What month is it?</u>

Incorrect	0
Correct	1

29. {DAY}

<u>What day of the week?</u>

Incorrect	0
Correct	1

30. {YEAR}

What year is it?

	Incorrect	0
	Correct	1 (within one year)

31. {SEASON}

What season is it?

	Incorrect	0
	Correct	1 (appropriate to the setting)

(Wet or dry were the appropriate alternatives in Nigeria)

I am going to ask you to carry out some actions so please listen carefully, because I will only tell you one time
(Interviewer -, give complete instructions at one time, do not give step by step)

32. {NOD}

Please nod your head

	Incorrect	0
	Correct	1

33. {POINT}

Please point first to the desk and then to the door

	Incorrect	0
	Correct	1

34. {PAPER}

I'm going to give you a piece of paper. When I do, take the paper in your right hand, fold the paper in half with both hands, and put the paper down on your lap.

Score one point for each component carried out correctly

	Uses right hand	1
	Folds in two	1
	Places in lap	1

TOTAL SCORE _____ (maximum 3)

35. **Now I would like you to take my pencil and copy these figures in the space given below them on the sheet**

See figures on next two sheets

35.1 {CIRCLE}

Score for circles

	Incorrect	0
	Correct	1

Score one if two vaguely circular objects intersect to form a meniscus

35.2 {PENTAG}

Score for pentagons

Incorrect	0
Correct	1

Score one if two five sided objects intersect to form a diamond shape

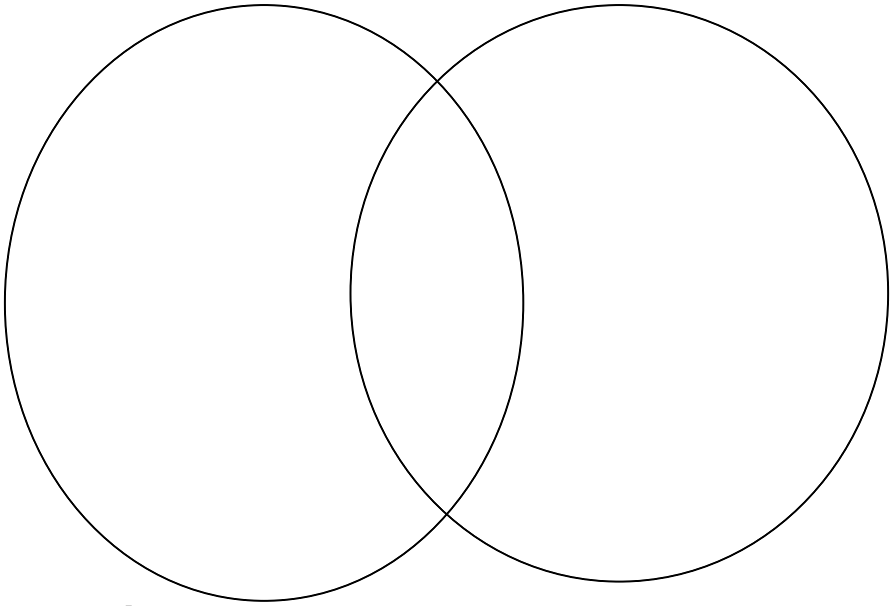

Copy here

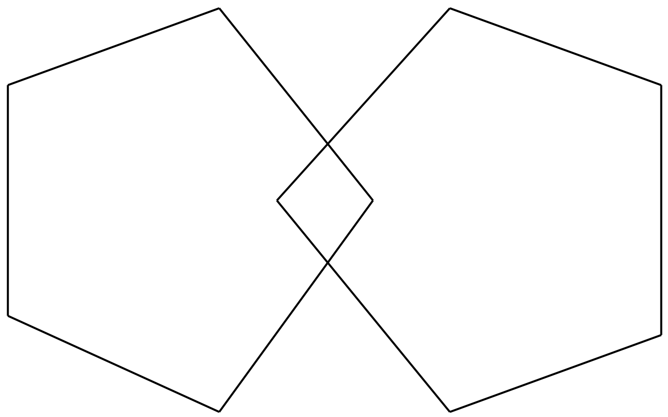

Copy here

Now I will tell a short story, then I will ask you to repeat as much of the story as you can remember. I want you to listen very carefully because I want you to try to tell me the whole story with as many details as you can remember.

Three children were alone at home and the house caught on fire. A brave man managed to climb in a back window and carry them to safety. Aside from minor cuts and bruises, all were well.

Now I would like you tell me the story in as much detail as possible

36. {STORY}
Story recall – total items recalled
Interviewer - score one point for each component correctly recalled

3 children	1
house on fire	1
brave man climbed	1
children rescued	1
minor injuries	1
everyone well	1

TOTAL SCORE ____ (maximum 6)

EURO-D scale

<u>We are interested in the kind of problems people may have and particularly the way you have been feeling over the past month. It is simply a study of people's possible problems.</u>

<u>Don't be concerned if some questions appear a little odd or strange, some of them will not apply to you, but we have to ask everyone the same sort of questions.</u>

<u>All of the following questions refer to the last one month</u>

MH1 [GMS21]. DEPRESSION
<u>In the last month, have you been sad or depressed?</u>
1 Yes
2 No
QbyQ: If participant asks for clarification, say 'By sad or depressed, we mean miserable, in low spirits, or blue'

MH2. [GMS 29] PESSIMISM
<u>What are your hopes for the future?</u>
1 ANY hopes mentioned
2 NO hopes mentioned

MH3. [GMS30] WISHING DEATH
<u>In the last month, have you felt that you'd rather be dead?</u>
1 Any mention of suicidal feelings or wishing to be dead
2 No such feelings mentioned
QbyQ: This question asks about a specific wish, or preference to be dead. For participants who express ambivalence about living or dying code 2.
ANY wish or preference to be dead would be coded 1, even if the participant has felt this only occasionally in the last month

EXAMPLES
Code 1 Any mention of suicidal feelings or wishing to be dead
"Yes"
"Sometimes, I feel that, but not for long"
"Yes, I want to die now, my life is over"
"I'm looking forward to my death so I can see my husband again'
Code 2 No such feelings mentioned
"I don't really care if I die now, I feel I'm at the end of my life"
"I don't mind. I take each day as it comes"

MH4. [GMS104] GUILT
4.1 **<u>Do you tend to blame yourself or feel guilty about anything?</u>**

1 Obvious excessive guilt or self blame
2 No such feelings
3 Mentions guilt or self-blame, but it is unclear if these constitute obvious or excessive guilt or self blame

if 1 or 2 then go to MH5
if 3 then ask-

MH4.2 **<u>So, for what do you blame yourself?</u>**

1 Example(s) given constitute obvious excessive guilt or self blame
2 Example(s) do not constitute obvious excessive guilt or self blame, or it remains unclear if these constitute obvious or excessive guilt or self blame

QbyQ - Only code 1 for an exaggerated feeling of guilt, which is clearly out of proportion to the circumstances. The fault will often have been very minor, if it there was one at all. Justifiable or appropriate guilt should be coded 2.

EXAMPLES

<u>Obvious excessive guilt or blame (code 1)</u>
Unusually, very severely depressed people may have lost touch with reality e.g "The September 11th attack on the World Trade centre was my fault. I am to blame"
More commonly, depressed older people feel responsible in an exaggerated way for bringing harm or hurt to those around them
"I am a burden on my children. I am useless and just hold them back"

<u>Trivial, justifiable or appropriate guilt (code 2)</u>
"I left my wife and haven't seen much of my children. I blame myself for this"
"I have not been as kind or considerate to people as I should"

MH5. [GMS54] SLEEP

<u>Have you had trouble sleeping recently?</u>

1 Trouble with sleep or recent change in sleep pattern
2 No trouble sleeping

QbyQ - Any trouble sleeping is coded here. Specifically, sleep problems attributed to need to get up to pass water, bodily pain or discomfort, or noisy environment should still be coded 1

MH6. [GMS113] INTEREST

6.1 **<u>In the last month, what is your interest in things?</u>**

1 Less interest than is usual mentioned
2 No change in levels of interest mentioned
3 Non-specific or uncodeable response

if 1 or 2 then go to MH7
if 3 then ask-

MH6.2 **<u>So, do you keep up your interests?</u>**

1 No
2 Yes

MH7.[GMS105] IRRITABILITY

Have you been more irritable recently?
1 Yes
2 No

MH8 [GMS51]. APPETITE
8.1 **What has your appetite been like?**
1 Diminution in the desire for food
2 No diminution in the desire for food
3 Non-specific or uncodeable response
QbyQ - It is APPETITE (desire for food) that is being asked about here. Someone who is eating less because of a diet, but does not have any 'diminution in the desire for food' should be coded 1

if 1 or 2 then go to MH9
if 3 then ask-

MH8.2 **So, have you been eating more or less than usual?**
1 less
2 more
3 neither more nor less
QbyQ – Again, someone who volunteers that they are eating less than usual, but because of a deliberate diet should be coded 3

MH9. [GMS72] FATIGUE
In the last month, have you had too little energy to do the things you want to do?
1 Yes
2 No

MH10 [GMS117/118]. CONCENTRATION
10.1 [GMS117] **How is your concentration? For example, can you concentrate on a television programme/ film/ radio?**
1 Difficulty in concentrating on entertainment mentioned
2 No such difficulty mentioned
QbyQ – Participants who are blind should just be asked about concentrating on a radio programme. Other should be asked about 'a television programme, a film or a radio programme'

MH10.2 [GMS 118] **Can you concentrate on something you read?**
1 Difficulty in concentrating on reading mentioned
2 No such difficulty mentioned
QbyQ – For those who cannot read because of blindness or illiteracy, a missing value code will be entered

MH11. [GMS114] ENJOYMENT
What have you enjoyed doing recently?
1 Fails to mention any enjoyable activity

2 Mentions ANY enjoyment from activity

MH12 [GMS22]. TEARFULNESS

<u>In the last month, have you cried at all?</u>

1 Yes

2 No

QbyQ – Any episode of crying would be coded 1 ‘yes’, no matter what the cause. For example, crying over a scene in a film. Do check (if it is unclear from their answer) that the participant is describing crying IN THE LAST MONTH. If necessary, you should repeat the question, stressing this element.

10/66 Dementia Research Group
Population-based study
May 2003

Informant questionnaire

(CSI-D component)

INFORMANT INTERVIEW

NOTES FOR INTERVIEWER

1) Who should be the informant?

The informant should be the person who knows the older person best.
They are likely to be coresident with the older person, but do not have to be, if a non-coresident is better qualified to be the informant.
They are likely to be a family member, but again, do not have to be, if a friend or neighbour is better qualified to be the informant.
Time spent with the older person may be a criteria for deciding the best informant, if there are several coresident family members.
Where the older person needs care and support, then you should aim to select the main caregiver as the informant. The main caregiver may be the person who provides most 'hands on' care, or may be the family member who organises the arrangements for 'hands on' care. In general, you should not select a paid caregiver to be the informant; choose the family member who is the main organisational caregiver instead.

2) Content

The informant interview has three modules

MODULE 1 BACKGROUND MODULE

This module provides information about the informant, and is administered, in full, to all informants

MODULE 2 CARE MODULE

This module provides information about care arrangements for the older person, and impact upon the caregiver. It is administered only when the older person has at least some care needs. Otherwise, after an initial screen you skip to Module 3.

MODULE 3 INFORMANT MODULE

This module provides clinical information about the older person, from the perspective of the informant.
The informant section of the Community Screening Instrument for Dementia informant (asking about evidence of cognitive and functional impairment) is administered in full to all informants.
The longer HASDDS (asking in detail about the course and onset of a possible dementia syndrome) is only administered if there are at least some signs of cognitive or functional impairment noted in the CSI-D. Otherwise you skip to the NPI-Q
The NPI-Q (asking about behavioural and psychological symptoms) is administered in full to all informants

MODULE 3 INFORMANT MODULE

Now I am going to ask you some questions about xxxx. As you know, your xxxx has given me permission to speak to you about him/ her. It is important to have another point of view, as we all find it difficult to describe ourselves.

1. COGNITIVE AND FUNCTIONAL IMPAIRMENT
(CSI-D: Section B - Informant interview)

FIRST OF ALL, I WOULD LIKE TO ASK A FEW BRIEF QUESTIONS ABOUT HER ACTIVITIES NOWADAYS.

I.CSID.1 **HAVE YOU SEEN A CHANGE IN HER DAILY ACTIVITIES IN THE PAST SEVERAL YEARS?**
IF YES - **PLEASE DESCRIBE**
0 No change
1 Diminution in range of activities, and/ or reduced ability to carry out activities
{ACTIV} #

I.CSID.2 **HAS THERE BEEN A GENERAL DECLINE IN HER MENTAL FUNCTIONING?**
0 No change
1 General decline
{MENTAL} #

I.CSID.3 **WE ALL HAVE SLIGHT DIFFICULTIES IN REMEMBERING THINGS AS WE GET OLDER, BUT HAS THIS BEEN A PARTICULAR PROBLEM FOR HER?**
0 No
1 Yes
{MEMORY} #

NOW I WOULD LIKE TO ASK YOU ABOUT OTHER CHANGES YOU MAY HAVE NOTICED IN HER

I.CSID.4 **DOES SHE FORGET WHERE SHE HAS PUT THINGS?**
IF YES – **HOW OFTEN DOES THAT HAPPEN?**
0 No
1 Yes, Sometimes
2 Yes, Regularly
{PUT} #

I.CSID.5 **DOES SHE FORGET WHERE THINGS ARE USUALLY KEPT?**
IF YES – **HOW OFTEN DOES THAT HAPPEN?**
0 No
1 Yes, Sometimes
2 Yes, Regularly
{KEPT} #

I.CSID.6 **DOES SHE FORGET THE NAMES OF FRIENDS?**
IF YES – **HOW OFTEN DOES THAT HAPPEN?**
0 No
1 Yes, Sometimes
2 Yes, Regularly
{FRDNAME} #

I.CSID.7 **OR MEMBERS OF THE FAMILY?**
IF YES – **HOW OFTEN DOES THAT HAPPEN?**
0 No
1 Yes, Sometimes
2 Yes, Regularly
{FAMNAME} #

I.CSID.8 **DOES SHE FORGET WHAT SHE WANTED TO SAY IN THE MIDDLE OF THE CONVERSATION?**
IF YES – **HOW OFTEN DOES THAT HAPPEN?**
0 No
1 Yes, Sometimes
2 Yes, Regularly
{CONVERS} #

I.CSID.9 **WHEN SPEAKING DOES SHE HAVE DIFFICULTY SAYING THE RIGHT WORDS?**
IF YES – **HOW OFTEN DOES THAT HAPPEN?**
0 No
1 Yes, Sometimes
2 Yes, Regularly
{WORDFIND} #

I.CSID.10 **DOES SHE USE THE WRONG WORDS?**
IF YES – **HOW OFTEN DOES THAT HAPPEN?**
0 No
1 Yes, Sometimes
2 Yes, Regularly
{WORDWRG} #

I.CSID.11 **DOES SHE TEND TO TALK ABOUT WHAT HAPPENED LONG AGO RATHER THAN THE PRESENT?**
IF YES – **HOW OFTEN DOES THAT HAPPEN?**
0 No
1 Yes, Sometimes
2 Yes, Regularly
{PAST} #

I.CSID.12 **DOES SHE FORGET WHEN SHE LAST SAW YOU?**
IF YES – **HOW OFTEN DOES THAT HAPPEN?**
0 No
1 Yes, Sometimes
2 Yes, Regularly
{LASTSEE} #

I.CSID.13 **DOES SHE FORGET WHAT HAPPENED THE DAY BEFORE?**
IF YES – **HOW OFTEN DOES THAT HAPPEN?**
0 No
1 Yes, Sometimes
2 Yes, Regularly
{LASTDAY} #

I.CSID.14 **DOES SHE FORGET WHERE SHE IS?**
IF YES – **HOW OFTEN DOES THAT HAPPEN?**
0 No
1 Yes, Sometimes
2 Yes, Regularly
{ORIENT} #

I.CSID.15 **DOES SHE GET LOST IN THE COMMUNITY?**
IF YES – **HOW OFTEN DOES THAT HAPPEN?**
0 No
1 Yes, Sometimes
2 Yes, Regularly
{LOSTOUT} #

I.CSID.16 **DOES SHE GET LOST AT HOME, E.G FINDING THE TOILET?**
IF YES – **HOW OFTEN DOES THAT HAPPEN?**

0 No
1 Yes, Sometimes
2 Yes, Regularly

{LOSTIN} #

I.CSID.17 **DOES SHE HAVE DIFFICULTY PERFORMING HOUSEHOLD CHORES THAT SHE USED TO DO, SUCH AS PREPARING FOOD OR BOILING A POT OF TEA?**
IF YES - **HOW OFTEN DOES THAT HAPPEN?**
0 No
1 Yes, Sometimes
2 Yes, Regularly

{CHORES} #

I.CSID.17a Does the INTERVIEWER think that the problem is primarily due to physical disability?
0 No, not due to physical disability
1 Yes, due to physical disability

{CHOREDIS} #

I.CSID.18 **HAS THERE BEEN A LOSS OF A SPECIAL SKILL OR HOBBY SHE COULD MANAGE BEFORE?**
0 No
1 Yes

{HOBBY} #

I.CSID.19 **HAS THERE BEEN A CHANGE IN HER ABILITY TO HANDLE MONEY?**
0 No difficulty
1 Some difficulty
2 Can not handle money

{MONEY} #

I.CSID.20 **DOES SHE HAVE DIFFICULTY IN ADJUSTING TO CHANGE IN HER DAILY ROUTINE?**
IF YES - **HOW OFTEN DOES THAT HAPPEN?**
0 No
1 Yes, Sometimes
2 Yes, Regularly

{CHANGE} #

I.CSID.21 **HAVE YOU NOTICED A CHANGE IN HER ABILITY TO THINK AND REASON?**
0 No
1 Yes

{REASON} #

I.CSID.22.1 **DOES SHE HAVE DIFFICULTY FEEDING HERSELF?**
0 Eats cleanly with proper utensils
1 Eats messily with a spoon only
2 Simple solids such as biscuits
3 Has to be fed

{FEED} #

I.CSID.22.2 Does the INTERVIEWER think that the problem is primarily due to physical disability?
0 No, not due to physical disability
1 Yes, due to physical disability

{FEEDDIS} #

I.CSID.23.1 **DOES SHE HAVE DIFFICULTY DRESSING?**
0 Dresses self
1 Occasionally misplaces buttons etc.

2 Wrong sequences, commonly forgets items
3 Unable to dress

{DRESS} #

I.CSID.23.2 Does the INTERVIEWER think that the problem is primarily due to physical disability?
0 No, not due to physical disability
1 Yes, due to physical disability

{DRESSDIS} #

I.CSID.24.1 **DOES SHE HAVE DIFFICULTY USING THE TOILET? DOES SHE WET OR SOIL HERSELF?**
0 No problems
1 Occasionally wets bed
2 Frequently wets bed
3 Double incontinence

{TOILET} #

I.CSID.24.2 Does the INTERVIEWER think that the problem is primarily due to physical disability?
0 No, not due to physical disability
1 Yes, due to physical disability

{TOILDIS} #

I.CSID.25 **DOES SHE EVER MISTAKE YOU (OR OTHER FAMILY OR FRIENDS) FOR SOMEONE ELSE?**
0 No
1 Yes

{MISTAKE} #

I.CSID.26 **HAS SHE FOUND DIFFICULTY IN MAKING DECISIONS RECENTLY ABOUT EVERYDAY THINGS**
0 No
1 Yes

{DECIDE} #

I.CSID.27 **DOES HER THINKING EVER SEEM MUDDLED?**
0 No
1 Yes

{MUDDLED} #
