# Supplementary material for: Development of a brief assessment and algorithm for ascertaining dementia in low-income and middle-income countries: the 10/66 short dementia diagnostic schedule
Source: BMJ Open. 2016 May 25;6(5):e010712. doi: 10.1136/bmjopen-2015-010712 (PMC4885443; doi:10.1136/bmjopen-2015-010712)
Supplement: Supplementary appendix 2 [file bmjopen-2015-010712supp2.pdf]

Appendix 2: SPSS syntax for 10/66 short-form predictive algorithm generation

```
RECODE
  nrelscor
  (1=0) (2=1.497) (3=2.251) (4=4.343) (5=6.088) INTO xrelscor .
EXECUTE .

RECODE
  ncogscor
  (1=3.792) (2=2.208) (3=1.174) (4=-0.048) (5=0) INTO xcogscor .
EXECUTE .

RECODE
  ndelay
  (5=0) (4=1.500) (3=1.721) (2=2.454) (1=3.241) INTO xdelay .
EXECUTE .

RECODE
  euro_group
  (0=0) (1=0.576) (2=-0.312) (3=-1.214) INTO xeuro .
EXECUTE .

COMPUTE logodds_short = -8.905 + xrelscor + xcogscor + xdelay + xeuro .
EXECUTE .

COMPUTE odds_short = EXP(logodds_short) .
EXECUTE .

COMPUTE prob_short = odds_short/(1+odds_short) .
EXECUTE .

RECODE
  prob_short
  (Lowest thru 0.199999999999=0) (0.20 thru Highest=1) INTO dem1066_short .
EXECUTE .
```

Where ‘nrelscor’, ‘ncogscor’, ‘ndelay’ and ‘euro_group’ are the categories defined in the second column of Table 1.
